# Kinase overexpressing cancers responsive to drug withdrawal

**DOI:** 10.18632/aging.100830

**Published:** 2015-10-22

**Authors:** Amit Dipak Amin, Soumya S. Rajan, Jonathan H. Schatz

**Affiliations:** Department of Medicine, Division of Hematology‐Oncology, Sylvester Comprehensive Cancer Center, University of Miami Miller School of Medicine, Miami, FL, 33136

**Keywords:** oncogene overdose, ALK, BRAF, cancer therapeutics

Aberrant protein kinase activity promotes tumor survival and proliferation, and targeted kinase inhibitors that halt growth and promote apoptosis demonstrate some cancers are truly kinase addicted. Clinically, this is best exemplified by chronic myeloid leukemia (CML), driven by the fusion kinase BCR-ABL, where tyrosine kinase inhibitor (TKI) therapy can control the disease for years, perhaps indefinitely in many patients. For other cancers, however, the success of kinase inhibition has been more modest. Despite great strides in drug design and delivery, resistance invariably develops, typically limiting median progression free survival (PFS) to a period of months. Development of new-generation inhibitors therefore has focused on increasing potency, overcoming resistance-conferring mutations to the drug target, and hitting parallel signaling pathways that bypass the target altogether. While sequential treatments and/or combination cocktails to circumvent resistance may work in some cases, concerns arise regarding toxicity and cost, prompting exploration of innovative new strategies to prolong PFS. Two recent studies in different cancers propose an alternative with a potential to increase the duration of tumor control by several already approved TKIs.

Approximately 70% anaplastic large cell lymphoma (ALCL) cases are driven by the constitutively activated fusion kinase NPM-ALK [1]. To investigate resistance mechanisms, our laboratory grew patient-derived NPMALK-driven cell lines in one of two FDA approved ALK TKIs, crizotinib or ceritinib, at increasing concentrations. Resistance reliably arose due to overexpression of NPM-ALK even if resistanceconferring mutations also began to arise. Strikingly, characterization of resistant phenotypes showed viability of these cells was actually stimulated by – indeed required – continued presence of ALK TKI, as drug withdrawal rapidly induced apoptosis. Concomitantly we observed massive ALK activation, suggesting over-activation provides as much of a fitness deficit as inhibition [2]. These results echo the findings of a prominent study investigating mutant-BRAF inhibition with vemurafenib in melanoma, where resistance also arose due to target overexpression [3]. As in our study, resistant cells underwent apoptosis in response to inhibitor withdrawal. Oncogene over-expression in both reports therefore promoted a dual phenotype of drug resistance and dependence.

Both studies demonstrate potential therapeutic exploitation of the paradoxically toxic response of resistant-dependent cells to drug withdrawal. Xenografted resistant cells in both reports underwent apoptosis leading to tumor regressions upon discontinuation of drug dosing to host animals. After time tumors resumed growth, but sampling showed drug-target expression had returned to baseline – a requirement for their growth without inhibitor. At the same time dependence went away, so did resistance, as re-initiation of inhibitor dosing to host animals led to new rounds of tumor regressions. This suggested cycling of drug through discontinuous dosing could forestall onset of fatal resistance, and was explored in both reports, but especially in the melanoma models [2, 3]. Here both pre-scheduled and individualized intermittent dosing strategies greatly prolonged tumor control compared to continuous drug administration. Patients at risk of developing resistance due to upregulation of some oncogenes may therefore benefit from intermittent dosing, a strategy carrying both low cost and low toxicity.

A randomized phase 2 trial comparing intermittent vs. continuous inhibitor dosing in melanoma already is enrolling patients (NCT02196181), and one is in planning for ALK+ ALCL. More preclinical assessment, however, also is needed in these and other cancers. Supporting the approach, drug holidays already may be employed to counter toxicity, and some second remissions to the ALK TKI crizotinib have been reported in lung cancer patients whose tumors were previously resistant [4, 5].

Great care must be taken, when determining appropriate timing of drug administration and withdrawal with such strategies, as the onset of resistance may be unpredictable, and drug interruption or re-initiation too early could exacerbate the onset of other resistance pathways [6]. An intriguing alternative, however, is identifying the specific mechanisms by which over-activity of particular oncogenes promote toxicity, and then pharmacologically inducing them as either a direct means of cellular toxicity or to prime the cells for other therapies. Indeed, a recent study showed pharmacological induction of SYK hyper-activation caused BCR-ABL+ acute lymphoblastic leukemia (ALL) cell death [7]. Targeted inhibition of several signaling pathway targets downstream failed to rescue resistant cells from NPM-ALK overdose in our systems (unpublished observations), but unbiased approaches are ongoing to determine the mechanisms.

Finally, it is important to keep in mind that intermittent dosing is not a cure and drug cycling eventually will prove futile. Such strategy gives a patient more time, however, delaying the need to change inhibitors, initiate combination cocktails, or a move to traditional therapies like chemotherapy or radiation, all of which may be significantly more toxic. In the ever-expanding arsenal of weapons against cancer, strategies exploiting oncogene overdose appear to hold promise, and in the case of intermittent dosing don't even require development of new drugs.
